# Traumatic spigelian hernia complicated by caecal perforation and retroperitoneal haematoma—an unusual concurrence in blunt trauma

**DOI:** 10.1093/jscr/rjad003

**Published:** 2023-01-18

**Authors:** Faisal Syed, Gaik si Quah, Angelina Di Re

**Affiliations:** Department of Surgery, Dubbo Base Hospital, Dubbo, New South Wales, Australia; Department of Surgery, Dubbo Base Hospital, Dubbo, New South Wales, Australia; Department of Surgery, Dubbo Base Hospital, Dubbo, New South Wales, Australia

## Abstract

Blunt abdominal injury can be a cause of significant morbidity and mortality in patients who have sustained trauma even at low velocity. We report on an unusual case of a 50-year-old male who developed a traumatic spigelian hernia and caecal perforation after falling off a pushbike and impacting on the handlebar. We have also discussed some of the considerations taken in the assessment and surgical management of this case.

## INTRODUCTION

Blunt abdominal injury and the concurrent increase in intra-abdominal pressure can result in bowel or abdominal organs to herniate through a disruption of abdominal musculature or fascia [1, 2]. Known as traumatic abdominal wall hernias (TAWH), such hernias can occur anywhere and even away from the site of injury at a location of anatomical weakness. While mesenteric injury and bowel injury are worrisome in blunt abdominal trauma, concurrent traumatic herniation and bowel perforation is a rare phenomenon.

## CLINICAL SCENARIO

A 50-year-old male presented as a trauma to the Emergency Department (ED) after falling off a pushbike at low speed and forcefully landing on the end of the handlebar resulting in a painful lump to the right lower quadrant. The lump was initially small at the time of injury; however it had expanded in size on examination in ED. It was soft, but tender and irreducible with overlying abrasions. On arrival he was mildly tachycardic with a borderline blood pressure (Table 1) that responded well with fluid resuscitation. Haemodynamics were eventually normalized, and no other injuries were identified on primary and secondary survey. He had a history of chronic back pain and opiate use, and recent amphetamine use but there was no history of abdominal hernias, previous surgeries or any other medical history. Blood results on presentation are provided in Table 2.

**Table 1:** Vital signs at the time of presentation to ED.

Pulse	95 p/m
Blood pressure	100/60
Temp	37.2
Pain score	8/10
Respiratory rate	18
O^2^ saturation	93%
News score	5

**Table 2:** Bloods investigation at the time of presentation to ED.

Haemoglobin	137	NR: 130–180 g/L
White cell count	4.9	NR: 3.7–9.5 × 10*9/L
Platelets	224	NR: 150–400 × 10*9/L
Neutrophils	2.2	NR: 2.0–8.0 × 10*9/L
Sodium	138	NR: 135–145 mmol/L
Potassium	3.5	NR: 3.2–5.0 mmol/L
eGFR	>90	NR: 60–90 ml/min
Urea	3.0	NR: 3.0–7.5 mmol/L
AST	13	NR: <40 u/L
ALT	26	NR: <40 u/L
Bilirubin	5	NR: <40 umol/L

**Figure 1:**
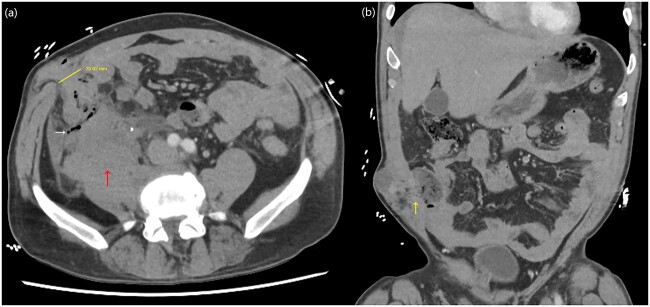
Abdominal CT axial (**a**) and coronal slices (**b**) demonstrating a 25 mm defect in the anterior abdominal wall lateral to rectus abdominis in the right lower quadrant containing caecum with adjacent small locules of extraluminal gas as well as a right-sided retroperitoneal haematoma.

**Figure 2:**
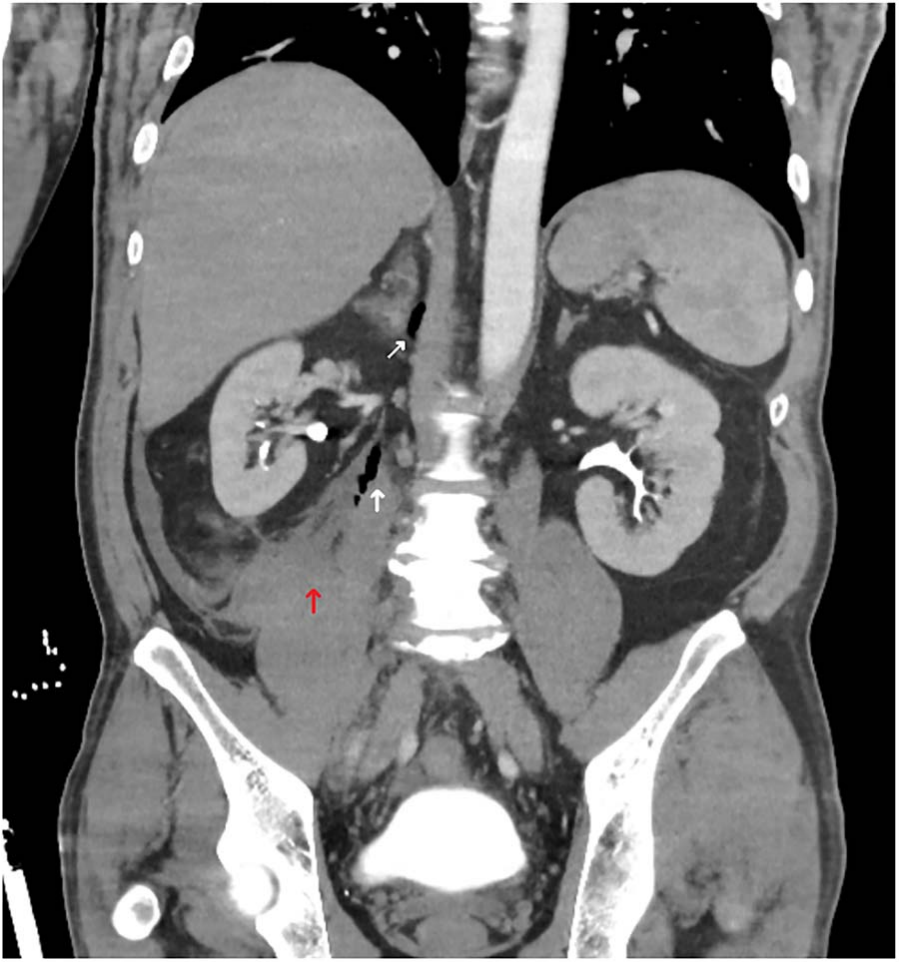
Abdominal CT coronal slice demonstrating a right-sided retroperitoneal haematoma with gas locules tracking up towards the right adrenal.

A computed tomography (CT) trauma series scan of his abdomen (Fig. 1) revealed an acute traumatic spigelian hernia through a 25 mm defect containing his caecum as well as caecal bruising and free fluid in the right paracolic gutter. He was also found to have an estimated 500 mL retroperitoneal haematoma anterior to the right psoas with overlying gas (Fig. 2). Given the suspicion for bowel perforation secondary to the acute traumatic spigelian hernia, emergency surgery was immediately organized.

In the emergency laparotomy, the findings were caecal perforation with faecal contamination extending into the right retroperitoneum and the associated haematoma. A limited right hemicolectomy from terminal ileum to hepatic flexure was performed with a side-to-side functional end-to-end stapled anastomosis. The abdomen was copiously irrigated, and a drain was inserted into the retroperitoneal defect. The spigelian hernia was closed primarily in two layers with 1.0 nylon suture. Post-operative recovery was complicated by pain and ileus but the patient made an eventual recovery with supportive, non-operative management and was discharged home on Day 10.

## DISCUSSION

First described by Selby in 1906, a traumatic spigelian hernia occurs as a result of a defect at the semilunar line, lateral to the rectus abdominis muscle [3, 4]. Given that TAWH occur in <1% of all blunt abdominal injury and that spigelian hernias generally have a natural incidence of up to 2%, a traumatic spigelian hernia that contains bowel loops or ruptured bowel is a relatively rare phenomenon [4, 5].

In our case presentation, there was a high index of suspicion for a TAWH based upon clinical history and examination findings despite the low velocity of injury. An unusual finding was the depth of injury extending to the retroperitoneum and towards the right adrenal, suggesting that focal blunt trauma can manifest similar to a penetrating injury. This case highlights the advantage of CT in examining for additional traumatic intra-abdominal or retroperitoneal injuries, thus reinforcing CT as the modality of choice for investigating TAWH and their immediate complications [4, 6]. Although a FAST scan had not been performed for this patient, ultrasonography is unreliable in examining for retroperitoneal or bowel injuries [7]. CT is also useful for surgical planning as uncomplicated TAWH with no other detected intra-abdominal injuries on radiology can be repaired with a direct open or laparoscopic approach, thus avoiding laparotomy and its associated morbidity [8].

However in an emergency setting, laparotomy is the recommended approach for complicated TAWH as up to 50% of TAWH cases are likely to have other intra-abdominal injuries such as mesenteric or bowel injury requiring surgery [9]. Closure of TAWH defects can either occur primarily or with mesh [4]. Primary closure is preferable in small tension-free defects or if there is contamination or hollow-viscus injury such as in this case, whereas mesh can be considered for larger defects or in a relatively clean operative field [4, 6].

### Learning points

(i) Blunt abdominal injury can cause disruption of the abdominal wall resulting in traumatic hernias.
(ii) Focal blunt abdominal trauma can potentially have a deep zone of injury and manifest similar to a penetrating injury.
(iii) An expanding mass in the context of abdominal trauma is concerning for vascular or intra-abdominal injury. Further imaging is useful in determining appropriate surgical intervention.
(iv) CT is the imaging modality of choice to investigate for traumatic abdominal wall hernias and identifying potentially complicating factors.
(v) Mesh reinforcement can be considered in the repair of traumatic abdominal wall hernias that are not contaminated.
